# The influence of the five-factor model of personality on performance in competitive sports: a review

**DOI:** 10.3389/fpsyg.2023.1284378

**Published:** 2023-12-14

**Authors:** Ying Shuai, Shaoshen Wang, Xian Liu, Yee Cheng Kueh, Garry Kuan

**Affiliations:** ^1^School of Sports Management, Shandong Sport University, Shandong, China; ^2^Unit of Biostatistics and Research Methodology, School of Medical Sciences, Universiti Sains Malaysia, Kota Bharu, Malaysia; ^3^Exercise and Sports Science Programme, School of Health Sciences, Universiti Sains Malaysia, Kota Bharu, Malaysia

**Keywords:** personality, five-factor model, sport performance, systematic review, competitive sports

## Abstract

Personality is considered to be a factor affecting athletic performance. However, inconsistency in the research results regarding size and even direction of the relationship. An evaluation of the evidence of the relationship between personality and athletic performance was conducted in order to summarize the evidence available. A systematic literature search was conducted in March 2023. Sport performance and the Big Five personality model were identified in our research. We used PubMed, Web of Science, Embase, Cochrane Library, Wang Fang (Chinese), Wei Pu (Chinese), and CNKI (Chinese) databases for the systematic literature search (Prospero registration number: CRD42022364000), screened 4,300 studies, and found 23 cross-sectional studies eligible for inclusion in this review. The results of this systematic analysis show that, besides neuroticism, openness, conscientiousness, extraversion, and agreeableness are all positively correlated with sports performance. Conscientiousness and extraversion are the two main personalities in team sports. Openness and agreeableness show different results in different sports, and it is not clear to which project they are beneficial. The value of personality as a possible predictor of athletic performance is generally positive. Therefore, professionals such as applied sports psychologists, coaching personnel, athletes, and sports administrators must comprehensively grasp the significance of personality’s role in achieving success in major competitions. Considering these facts, sports practitioners should promote personality screening and personality development programs.

## Introduction

The complex interplay between personality and sport has captivated the attention of researchers, coaches, and athletes for decades. Central to this discourse are two conflicting perspectives: the skeptical view, which argues that personality has a minimal effect on athletic ability, and the gullible perspective, which asserts a substantial influence of personality traits on sports performance. Recent studies, however, have introduced more nuanced theories, aiming to capture the multifaceted nature of this relationship. A comprehensive meta-analysis by Sutin et al. (2016) stands as a testament to this, offering compelling evidence regarding the association between personality and physical activity patterns. The study revealed that individuals with higher neuroticism levels were inclined toward inactivity, while those possessing elevated levels of extraversion, openness, and conscientiousness displayed reduced inactivity. These correlations remained significant, even when considering the shared variance between these traits. Remarkably, factors like age and sex did not alter these relationships, emphasizing the significant role of inherent tendencies in determining one’s engagement in physical activity. From the skeptical viewpoint, Davis, when assessing the personality traits of elite hockey players, found no correlation with their performance levels (Davis, 1991). In contrast, a large volume of literature spanning thousands of articles highlights the significance of personality within sports. Yet, it’s crucial to note that many of these studies employ different personality theories and testing methods, adding complexity to the interpretation of findings. For instance, an analysis of 42 British athletic groups’ mean scores on Cattell’s 16-PF questionnaire shed light on how personality traits might impact athletic performance. Interestingly, while top athletes showed lower anxiety levels than their less skilled peers, their anxiety was still above the population average, challenging the common notion that sports personalities are stable extraverts (Knapp, 1974). A crucial factor that differentiates one person from another is their unique personality (Karageorghis et al., 2021).

Indeed, while personality differences between individuals might appear subtle, their implications can be profound. Personality, as defined by Burger (2011), refers to consistent patterns of behavior and internal processes stemming from the individual. Historical work by E. W. Scripture at Yale University underscores the potential for cultivating certain personality traits through sports (Franz, 1898), emphasizing that sports success is, to a considerable extent, influenced by personality (Allen et al., 2013). Notably, Hartung and Farge’s assessment of middle-aged male runners revealed these athletes scored higher than the general populace in areas like intelligence, imagination, and self-sufficiency, among others (Hartung and Farge, 1977). Meanwhile, Cui Guofu’s research on elite Chinese race walkers found that introverted athletes outperformed extroverted ones, particularly among male participants (Cui, 1989).

In the sports domain, a longstanding question remains: is an athlete’s behavior primarily determined by the situation they find themselves in, or by their inherent personality? One intriguing study explored whether personality differences attracted individuals to sports, termed the “gravity hypothesis,” or if participation in sports molded an individual’s personality, the “developmental hypothesis.” Early findings suggested that team sport participants consistently scored higher on extroverted sport scales compared to individual sport participants and non-participants, thereby lending credence to the gravity hypothesis (Eagleton et al., 2007). Another significant theory to consider is the “performance hypothesis” posited by García-Naveira and Ruiz-Barquín (2013) (see also García-Naveira and Ruiz-Barquín, 2016; Garcia-Naveira and Ruiz-Barquín, 2020). This hypothesis emphasizes that certain personality traits are intrinsically linked with enhanced sports performance. It posits that these traits, to a certain degree, assimilate into the high-performance sports context, implying that athletes with these traits might naturally align better with the demands and rigors of elite sports competition. Instead of adhering to fixed notions of personality types, such as the Myers–Briggs typology, theories of person–environment fit (PEFT) advocate for a dimensional approach. This perspective views personality traits as influenced by and reflective of specific environments (Levy and Ruggieri, 2019).

Yet, the discussion extends beyond simply determining if personality affects sports participation and performance. It’s essential to highlight, as pointed out by multiple authors, including García Naveira (2010), García-Naveira et al. (2011), García-Naveira and Ruiz-Barquín (2016), and Piedmont et al. (1999), that personality’s influence is twofold. On one hand, it can directly affect the sporting action itself, and on the other, it can exert an indirect influence on the surrounding context and actions intimately tied to an individual’s sports performance. It’s crucial to discern the nature of this influence. Distinguishing between the direct and indirect effects of personality on sports behavior introduces additional complexity. A direct effect implies that personality traits linearly impact athletic performance. Conversely, an indirect effect suggests that personality traits shape other variables—like motivation or resilience—which subsequently affect sports outcomes. Allen et al. (2013) delved into this, investigating how personality shaped organized sports, its implications for athletic achievement, individual differences, and team dynamics. Their findings highlighted the interplay of both genetic and environmental factors, providing pivotal insights for applied sports psychology. Further exploring this theme, Allen and Laborde (2014) emphasized the predictive power of personality traits for both sports performance and broader physical activity. Their work uncovered connections between personality traits and various determinants, including the psychological state of athletes, harmful exercise behaviors, and even factors like strength and flexibility in older populations. Roberts and Woodman (2017) expanded the scope, focusing on the role of traits like narcissism and alexithymia in sports performance. Their call for an interactionist perspective underscores the intricate dance between personality and performance. Finally, a landmark study by Laborde et al. (2019) embarked on a sweeping overview of trait-based research in sports and exercise psychology. Analyzing a vast array of abstracts, they identified 64 unique traits clustered into 15 overarching themes, with traits like anxiety, self-efficacy, and perfectionism emerging as recurrent focal points. Their rigorous analysis linked many of these traits to the Big Five personality dimensions, although not all associations were straightforward.

Though many studies have explored the impact of personality in sports, it’s important to understand the diverse developmental trajectories of personality theory. Schools of personality development, such as psychoanalysis, behaviorism, social learning, cognitive theory, and humanistic theory, aim to elucidate how personality emerges and evolves over a lifetime (Cloninger, 2009). A vast body of literature, comprising thousands of articles, has examined the sports personality domain (Ruffer, 1976), with numerous investigations underscoring the significance of personality within the realm of sports. Each researcher used a different personality theory and test method, making the results difficult to analyze.

A prominent approach within psychological research, the trait or dispositional theory, seeks to quantify enduring patterns of behavior, cognition, and emotion termed as “traits” (Kassin, 2003). Rooted in this approach, the Big Five Personality Model emerged as a pivotal framework. Historically, by analyzing personality-related terms from dictionaries in the late 1920s, psychologists discovered the inherent structure of personality traits within our language. Factor analyses distilled these traits into five core factors: extraversion, agreeableness, conscientiousness, neuroticism, and openness to new experiences (Babcock and Wilson, 2020). The consistent appearance of these factors across studies led to the refinement of the Big Five Model (Goldberg, 1981).

According to this model, the terrain of human personality is primarily constituted by five central traits (Vealey, 2002; Allen et al., 2013; Gill et al., 2017):Neuroticism, defined by anxiety and tension vs. emotional stability. Extraversion, characterized by sociability and enthusiasm opposed to introversion. Openness, signified by adaptability and curiosity. Agreeableness, encompassing kindness and cooperativeness. Conscientiousness, marked by discipline and organization. This model posits that individual personalities are composed of varying degrees of these traits, with specific behaviors emerging as a result (McCrae and John, 1992). For instance, while conscientious individuals gravitate toward organization, neurotic individuals might be more self-conscious. The Big Five offers invaluable insights into personality differences, emphasizing the importance of individualized training approaches in sports (Rhodes et al., 2002).

Currently, sports psychologists are fervently exploring the Big Five traits (Lochbaum et al., 2010; Singley et al., 2012; Merritt and Tharp, 2013). Furthermore, they are examining additional traits like tolerance (Sheard and Golby, 2010) and their associations with athletes’ mental states and behaviors. Numerous studies have unveiled significant correlations between these traits, such as neuroticism and self-consciousness, and athletic performance (Piedmont et al., 1999; Wann et al., 2004). Allen et al. (2013) deduced from an extensive review that personality traits correlate with long-term athletic success. Their findings revealed that sports participants typically display greater extraversion than non-athletes. Moreover, team athletes in high-risk sports showed increased extraversion and reduced conscientiousness compared to those in lower-risk individual sports. As evidenced by Rhodes and Smith (2006), extraversion and conscientiousness positively influence physical activity, while neuroticism can serve as a deterrent.

The interaction between personality and sports performance, while multifaceted, undeniably influences the trajectory of sports success. From questioning the centrality of personality in determining athletic performance to drawing insights from the Big Five personality model, opinions vary, but there is a consensus that individual personality traits have a critical impact on athletic outcomes. In the pursuit of a comprehensive review, the objective is to address an evident lacuna in the extant literature. While there are five preceding reviews, the most recent one dating to 2019, a systematic exploration of the subject matter remains absent. Given the burgeoning research in this domain, a current and rigorous review is both relevant and imperative. By focusing on the Big Five personality traits, this review aims to utilize a standardized methodology to systematically assess their influence on sports performance. It is hypothesized that the Big Five traits significantly impact athletic performance, and the findings of this review will amalgamate the prevailing understanding of this association. Furthermore, this investigation will offer recommendations for future research, encompassing a variety of sports disciplines for comparative evaluation.

## Methods

The review was performed based on the Preferred Reporting Items for Systematic Reviews and Meta-Analysis (PRISMA) guideline (Page et al., 2021). Methods for conducting this review were pre-specified in a registered protocol on PROSPERO (CRD42022364000).

### Search strategy

A systematic literature search was conducted on PubMed, Web of Science, Embase, Cochrane Library, Wang Fang (Chinese), Wei Pu (Chinese), and CNKI (Chinese) databases in October until December 2022. The search queries were as follows: (“Athletic Performances” OR “Performance, Athletic” OR “Sports Performance” OR “Performance, Sports” OR “Performances, Sports” OR “Sports Performances”) AND (“Personalities” OR “temperament”). Two independent investigators searched databases, identified studies, screened them for eligibility, and compared them to each other. All related articles published from inception up to March 2023 were considered for inclusion.

### Inclusion and exclusion criteria

#### Inclusion criteria

Study type: cross-sectional studies examining the impact of published personality traits on athletic performance. Subjects: Athletes are assessed solely using the Big Five Personality Type Test, which comprises the Five-Factor Personality Model (FFM), a taxonomy of personality traits including conscientiousness, extraversion, openness, agreeableness, and neuroticism (OCEAN or NEOAC). All known personality traits are contained and encompassed within these five general domains, which are thought to represent the structure of all individual differences (O’Connor, 2002). There are no restrictions on gender or age. Exposure measures: the Big Five personality assessment. Outcome indicators: sports performance or performance within specific sports disciplines. Language limitation: Only studies published in Chinese or English, without regional restrictions, will be considered.

#### Exclusion criteria

Unspecified study type. Inability to extract valid outcome data from the text, absence of statistical analysis for impact results, improper application of statistical methods, or incomplete original data. Duplicate literature. Unavailability of the full text. Utilization of non-Big Five personality assessments. Insufficient sample size. In addition, letters, opinion articles, editorials, reviews, and papers that were not written in English were excluded from the review process. Animal, *in vitro*, *in vivo*, and modeling studies were also excluded.

### Study selection

**Figure 1:**
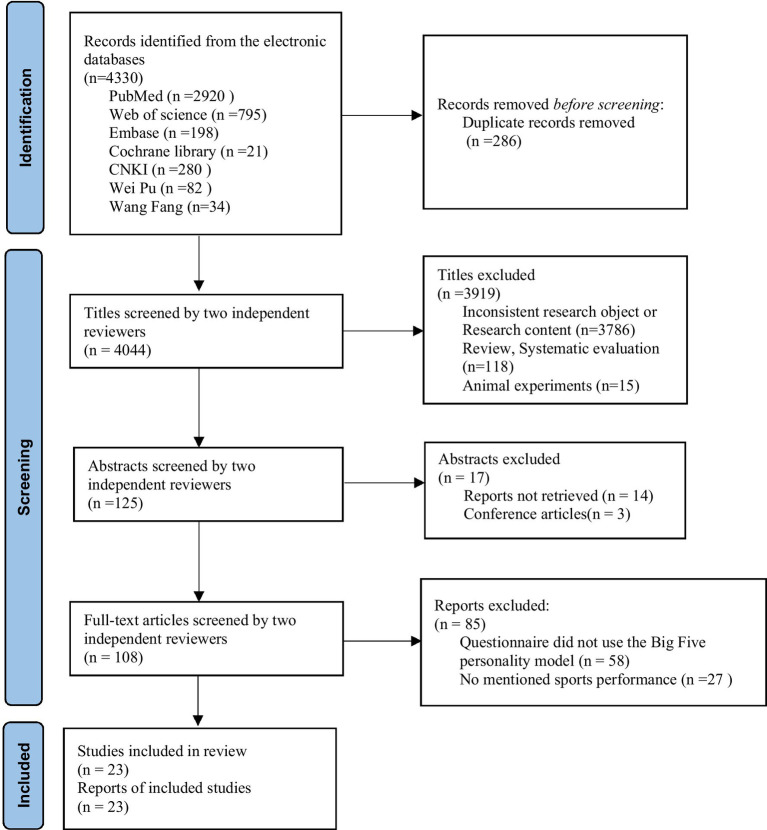
Flow chart of the search and selection process of included studies.

Two researchers individually screened titles and abstracts from the databases to determine eligibility using Endnote (version 20.0, Clarivate Analytics). They recorded the number of searches and duplicates for each database. Duplicate entries were eliminated using Endnote’s “check for duplicates” feature. Abstracts meeting the criteria underwent further screening to retrieve full-text articles. These articles were then evaluated against specific inclusion and exclusion standards. The researchers also conducted quality assessments and data extraction. Any disagreements were resolved by consulting a third investigator. References of the confirmed studies were manually checked. The article selection methodology is illustrated in Figure 1.

### Data extraction

Data was gathered using a standardized collection form. For every selected study, details such as the lead author’s surname, year of study, participant demographics, study design, sports disciplines, tools used, and outcomes were documented in Table 1. If any data was absent, the primary authors of those studies were directly approached for clarification.

**Table 1:** Basic information of included studies.

References	*N*	Age	Type of sport	Personality measurement	Measurement of sports performance	Other variables
Fabbricatore et al. (2021)	161 Male: 60.82% Female: 39.18%	*M* = 15.42 SD = 3.20 Range = 12–30	Swimming High level	Big Five dimensions in the Italian lexical context (Barbaranelli et al., 2007; Caprara and Perugini, 1994)	(P) considers race wins and participation, combining them into an increasing value scale with five anchored options, where 5 represents the best performance	Sport performance psychological inventory (IPPS-48) (Robazza et al., 2009)
Ionel et al. (2022)	272 Male: 158 Female: 114	*M* = 32.10 SD = 10 Range = 16–69	Rock-climbing Other level	Big Five Inventory–2 Short Form (BFI-2-S; Soto and John, 2017)	A single rock-climbing difficulty scale (IRCRA; Draper et al., 2015)	Grit was measured by employing the 12-item inventory (Duckworth et al., 2007)
Siemon and Wessels (2022)	185 Male: N/A Female: N/A	N/A	Basketball High level	IBM Watson Personality Insights Service (Ferrucci, 2012)	www.basketball-references.com www.sport-reference.com	N/A
Piedmont et al. (1999)	79 Male: 0 Female: 79	Range = 18–21	Soccer High level	Big five adjective marker scales (Piedmont, 1995)	Game statistics for each player were obtained from the most recent soccer season	Coaches ratings (Rosenthal and Rosnow,1984, p. 163)
Lin et al. (2011)	20 Male: 10 Female: 10	N/A	Canoeing High level	Big Five Personality Test (Costa and McCrae, 1992)	Chinese National Team Canoeing Performance at the 2008 Olympic Games	N/A
Liu (2011)	133 Male: 76 Female: 57	N/A	Difficult and beautiful events Fighting athletes Other level	NEO Five Factor Inventory (Xu et al., 1996)	Athletes’ competition performance: top three nationally, top three in Liaoning Province, others	Subjective well-being General Wellbeing Scale (GWB)
Allen et al. (2011)	253 Male: 187 Female: 66	M = 21.1 SD = 3.7 Range = 16–69	34 different sports High level	NEO-FFI (Costa and McCrae, 1992)	Different levels, including university, club, regional, national, and international	Coping Function Questionnaire for Sport (Kowalski and Crocker, 2001)
Zhu et al. (2013)	34 Male: N/A Female: N/A	M = 22.19 SD = 2.81 Range = 16–69	Boxing High level	Big Five Personality Inventory (Yao, 2010)	Athletes’ athletic performance was evaluated by their overall performance ranking in the National Boxing Championships, Boxing Championships and National Championships in 2011	Volitional quality (Yin, 1985) Mentalt tenacity (Li, 2009)
Terracciano et al. (2013)	642 Male: 52% Female: 48%	M = 61.07 SD = 12.86 Range = 31–96	Fast walking Non-athletes	NEO Personality Inventory (NEO-PI-R) (Costa & McCrae, 1992)	Metabolic Rate and Aerobic Capacity	N/A
Balyan et al. (2016)	50 Male:50 Female:0	M = 23.5 SD = 2.11 Range = 18–25	Computer-based soccer games Other level	Five Factor Personality Inventory (Tatar, 2005)	Play a computer-simulated soccer match against an experienced player (one of the experimenters) for 10 min	Competitive State Anxiety Inventory–2 (Martens et al., 1990) Physiological Arousal, Electrodermal activity (EDA)
Klein et al. (2017)	1,399 Male: 707 Female: 692	7th (12.9 ± 0.6) 10th (15.8 ± 0.6)	Motor performance Other level	NEO Personality Inventory (NEO-PI-R) (Costa and McCrae, 1992)	German motor performance test DMT (Deutscher Motorik-Test) 6–18 (Bös et al., 2009)	Physical self-concept (self-developed short scale)
Stine et al. (2019)	31 Male: 0 Female: 31	M = 20.3 SD = 1.2	Rower Other level	Neo FFI (five-factor inventory, version 3) (McCrae and Costa, 2004)	2,000-m Performance Tests Stroke rate, power output, and time to complete 2,000 m were recorded	N/A
Yuan (2020)	182 Male: 114 Female: 68	N/A	Routine of Martial Arts Other level	NEO Personality Inventory (NEO-PI-R) (Costa and McCrae, 1992)	Three evaluation indexes are chosen: single entrance exam technical score, competition award-winning score (32 grades, 3.125 points each, totaling 100 points), and self-technical evaluation	N/A
Kalinowski et al. (2020)	122 Male: 122 Female: 0	Range = 16–19	Soccer High level	Polish version of NEO Personality Inventory (Zawadzki et al., 1998)	Szwarc12, This study tool makes it possible to assess the play- ers’ effectiveness of performance in attack and defense by determining 15 effectiveness indicators (Szwarc, 2002)	Polish version of Coping Inventory for Competitive Sport CICS (Knittel and Guszkowska, 2016)
Matuszewski et al. (2020)	206 Male: 188 Female: 18	M = 19.99 SD = 1.88 Range = 18–27	Electronic sports High level Other level	NEO Personality Inventory (NEO-PI) (Costa and Mccrae, 1989)	League of Legends performance was operationalized here as position within the ranking ladder	N/A
Piepiora and Piepiora (2021)	1,260 Male: 1260 Female: 0	Range = 20–29	30 sporting disciplines High level	NEO-FFI Personality Inventory (Costa and Mccrae, 2007)	Champions and other athletes Sports achievements at various levels of rivalry (national, continental, and world). The best results of the respondents on the day of the study were included in the study	N/A
Zar et al. (2022)	376 Male: N/A Female: N/A	N/A	Disabled athletes team athletes High level	Big Five Personality Traits (Khormaee and Khayer, 2006)	Based on the information available in the provincial sports delegations and the Veterans and Disabled Federation, the positions obtained by each athlete were considered as a criterion for sports performance	N/A
Piepiora et al. (2021)	140 Male: N/A Female: N/A	Range = 20–29	American football Other level	NEO-FFI Personality Inventory (Costa and Mccrae, 2007)	Liga de Fútbol Americano Profesional LFA 1, LFA 2, and LFA 9	N/A
Piepiora (2021a)	300 Male: N/A Female: N/A	Range = 20–29	10 team sports High level Other level	NEO-FFI Personality Inventory (Costa and Mccrae, 2007)	Champions and other athletes sports. sports achievements at various levels of competition (national, continental, and world)	N/A
Klatt et al. (2021)	82 Male: 46 Female: 36	M = 26.39 SD = 4.32	Beach volleyball High level	German Big-Five-Inventory-10 (Rammstedt and John, 2007) Persönlichkeits-adjektiv-skalen (pask5) (Brandstätter, 2009)	Individual ranking points as an estimation for performance level	Affective Style Questionnaire (ASQ) (Graser et al., 2012)
Fasold et al. (2019)	84 Male: 84 Female: 0	M = 26.87 SD = 5.32	Handball High level	10-item short version of the Big Five Inventory in English and German (Rammstedt and John, 2007)	1st-3rd league vs. 4th league and lower playing in the 1st- 3rd league in Germany were considered as playing on a high performance level	N/A
Azita et al. (2019)	68 Male: N/A Female: N/A	Range = 18–22	Futsal Other level	The great five personality factors questionnaire (Costa and McCrae, 1987)	The researcher used a checklist to observe and record player performance during the game, which was then used to calculate the performance ratio for each sub-component	N/A
Ruiz-Barquín and García-Naveira (2013)	128 Male: 128 Female: 0	M = 17.5 SD = 2.5 Range = 14–24	Football High level Other level	Neo Personality Inventory NEO-FFI (Costa and McCrae, 2008)	Average athletic performance is evaluated by the coach using a 1–10 scale based on over 16 observations of each athlete’s league performance	N/A

M, mean; SD, standard deviation; N/A, no answer; N, participants; The sample numbers present the absolute sample size of the studies.

### Quality assessment

We used the STROBE (Strengthening the Reporting of Observational Studies in Epidemiology) Statement to evaluate the quality of reporting in each of the 15 cross-sectional studies. The STROBE Statement includes a checklist of 22 items that should be reported in observational studies. Each study was evaluated against each of the 22 items, and each item was scored as “Yes” (if the study reported the item), “No” (if the study did not report the item), or “Not Applicable” (if the item was not relevant to the study design). The STROBE Statement assesses key aspects of cross-sectional studies, including the title, abstract, introduction, methods, results, discussion, and other relevant information, to ensure transparent and comprehensive reporting (Page et al., 2021).

## Results

### Search result

The database search yielded a total of 4,330 articles, whereof 23 articles were finally included in this review (Figure 1) (Page et al., 2021).

## Eligibility of studies

Table 1 displays the particulars of the selected studies. Cross-sectional studies (references) adhered to the review criteria. All included studies received ethical approval from their respective institutions. According to the table, studies have examined how personality traits relate to athletic performance using the Five Factor Model (FFM). It provides information on the authors and year of publication, sample size (N) and demographics, type of sport, athlete level, personality measurement tools, sports performance metrics, and other variables assessed in each study. The studies encompass a diverse range of sports, such as swimming, rock climbing, basketball, soccer, canoeing, boxing, fast walking, computer-based soccer games, motor performance, rowing, and martial arts. Various instruments have been employed to measure the FFM, including the NEO Personality Inventory (NEO-PI-R), the Big Five Inventory-2 Short Form (BFI-2-S), and the IBM Watson Personality Insights Service. Additionally, the studies utilize different approaches to assess sports performance, such as race wins, competition rankings, and specific performance tests. Some of the investigations also examine other psychological variables, like grit, coping strategies, and subjective well-being.

Among the 23 studies incorporated in the analysis, the earliest publication dates back to 1999 (Piedmont et al., 1999), while the remaining studies were published between 2011 and 2022, with a peak of five articles in 2021 (Fabbricatore et al., 2021; Klatt et al., 2021), three of which were authored by Piepiora (Piepiora et al., 2021; Piepiora and Piepiora, 2021; Piepiora, 2021a). The included research encompasses four studies in Chinese and the rest in English. The investigations span an array of sporting disciplines, comprising individual and team events, fundamental physical fitness assessments, and innovative e-sports. Soccer research features prominently, with four articles dedicated to the subject. The studies encompass a broad age range, from 12 to 96 years, and extend beyond professional athletes to include the general population and individuals with disabilities. In many studies, personality characteristics have a certain impact on the performance of athletes in various sports. Characteristics such as openness, agreeableness, neuroticism, extraversion, and conscientiousness affect the performance of rock climbing, football, canoeing, boxing, and other sports to varying degrees. Moreover, an athlete’s personality traits can influence their emotional and physiological state leading up to a competition, which correlates with psychological aspects like anxiety and self-confidence. The well-being of athletes is related to their personality, and personality characteristics are also different among athletes of different ages. Generally speaking, personality characteristics have a certain reference value in the selection, training, and psychological adjustment of athletes.

Table 2 summarizes the key findings from the 23 reviewed articles, highlighting the outcomes related to the five unique personality traits: openness, conscientiousness, extraversion, agreeableness, and neuroticism, as observed across these studies. Though personality traits may not have a direct impact on swimming performance, they can indirectly influence it through mental skills (Fabbricatore et al., 2021). Openness and agreeableness have been linked with climbing performance (Ionel et al., 2022), while neuroticism and conscientiousness are associated with performance in female soccer players (Piedmont et al., 1999). Furthermore, personality traits have been demonstrated to predict the performance of elite Chinese rowers (Lin et al., 2011) and the subjective well-being of Chinese athletes in esthetically demanding and combat sports, with average ages of 14 and 18 years, respectively (Liu, 2011). The five-factor model of personality can distinguish varying levels of sports involvement and pinpoint the coping techniques adopted by athletes (Allen et al., 2011). In the elderly, there’s a notable link between personality traits (like neuroticism, extraversion, openness, and conscientiousness) and energy consumption. This suggests potential impacts on health aspects like weight management and lifespan (Terracciano et al., 2013). Neuroticism has been identified as crucial for comprehending athletes’ emotional and physiological states before competition due to its association with anxiety, arousal, and self-confidence (Balyan et al., 2016). Furthermore, personality attributes have been linked to performance outcomes in diverse sports, rowing being one of them (Stine et al., 2019), Chang Quan, Nan Quan, Taijiquan (Yuan, 2020), and young male soccer players (Kalinowski et al., 2020). In eSports, notable variances in traits like extraversion, agreeableness, and openness were detected between players at lower ranks and those at the higher tiers of the Legendary League (LoL) (Matuszewski et al., 2020). Sports champions typically exhibit lower neuroticism and higher scores in the other five dimensions, suggesting that neuroticism is a determining factor in the level of achievement.

**Table 2:** Summary of all studies examining Five Factor Model (FFM) and sports performance.

References	Main findings
Fabbricatore et al. (2021)	While personality traits do not have a direct significant impact on performance (as evidenced by the path coefficient results), their indirect effects, mediated through mental skills, and the cumulative impact (combining direct and indirect effects) are noteworthy for evaluation
Ionel et al. (2022)	The findings revealed that both openness and agreeableness were predictors of climbing performance. Additionally, grit had a more significant influence on climbing performance than the Five Factor Model (FFM) traits. While it’s a prevalent notion that grit and conscientiousness are synonymous, our results highlight that grit offers a distinctive role in explaining performance, especially in a relatively new and high-risk sport like climbing
Siemon and Wessels (2022)	The study validated a new methodology utilizing automated personality mining as a predictor of future basketball performance. This contribution advances the use of cognitive systems (automatic personality mining) and social media data for prediction. Scouts can use the results to improve their recruiting standards in the NBA enterprise
Piedmont et al. (1999)	This study highlights the significant associations between Neuroticism and Conscientiousness personality dimensions and athletic performance in female college soccer players. Results suggest that personality’s contribution to performance may be selective, with professional ratings offering valuable evaluative insights. The prototypical achiever’s personality profile consists of low Neuroticism and high Conscientiousness, indicating emotional stability and a drive to succeed. The findings also emphasize the potential to integrate sports research with the broader literature on motivation and performance, informing future research directions
Lin et al. (2011)	The results show that there are significant differences among the personality traits of elite canoeists in China. There are significant differences in some factors of personality traits among Chinese elite canoeists of different genders, ages, and training years. Some of the factors can predict the performance of Chinese elite canoeists
Liu (2011)	In Liaoning Province, China, active athletes tend to be emotionally sensitive and exhibit extroverted personalities, neither overly conservative nor excessively exploratory. No gender, regional, sports group, or training duration differences were observed in personality traits. Athletes in specialized improvement stages show lower agreeableness, while those in competitive maintenance stages exhibit higher agreeableness. As athletic performance improves, athletes demonstrate increased agreeableness. A correlation exists between athletes’ subjective well-being and their personalities, with personality effectively predicting athletes’ subjective well-being
Allen et al. (2011)	The five-factor model of personality appears to be a useful tool in distinguishing varying degrees of athletic participation and pinpointing coping mechanisms athletes might employ. Notably, there were distinct personality differences observed between elite and novice athletes, male and female competitors, as well as those engaged in individual vs. team sports
Zhu et al. (2013)	The results of a psychometric test with male boxers showed a significant correlation between the Neuroticism dimension of an athlete’s personality and performance
Terracciano et al. (2013)	This study investigated the association between personality traits and energy expenditure in older adults, finding that neuroticism, extraversion, openness, and conscientiousness were significantly related to energy expenditure during peak walking pace. These findings suggest that personality differences may play a role in health outcomes, such as obesity and longevity, particularly during more challenging activities that demand cardiorespiratory fitness
Balyan et al. (2016)	This research examined the relationship among personality traits, anxiety levels, and physiological responses in athletes. The findings highlighted a significant association between neuroticism and EDA when there were incentives involved and with different forms of anxiety within the group with higher anxiety levels. Athletes who won in this high anxiety group displayed increased cognitive anxiety but decreased physiological arousal compared to those who lost. In contrast, in the group with lower anxiety, no links were found between neuroticism, CSAI-2 elements, and physiological arousal
Klein et al. (2017)	This study discusses the relationship between personality traits and physical self-concept. Neuroticism is identified as a particularly influential trait, with lower emotional stability leading to a less positive view of one’s physical attractiveness and athleticism. The impact of reference groups, such as high-performance environments, is also discussed
Stine et al. (2019)	Neuroticism negatively impacts rowing performance, while agreeableness and conscientiousness trend toward better performance. Highly agreeable and conscientious rowers outperformed their less agreeable and less conscientious peers. The study suggests that personality traits may be important for athletic performance. However, limitations such as the small sample size and grouping based on personality traits call for further research
Yuan (2020)	The findings suggest that neuroticism negatively predicts performance in Changquan events, while conscientiousness positively predicts performance in Nanquan and Taichiquan events. Openness negatively predicts performance in Nanquan events. Strong willpower and anti-pressure ability are associated with better performance in Taichiquan and Changquan events, respectively. Additionally, personality traits have the greatest impact on performance in Nanquan events, followed by Changquan events, and then Taichiquan events
Kalinowski et al. (2020)	Lower neuroticism levels correlated with increased effectiveness through effort expenditure as a mediator. Higher conscientiousness levels led to greater performance effectiveness due to task-focused stress-coping strategies. Extraverted soccer players exhibited higher performance effectiveness by adopting task-focused methods for coping with stress
Matuszewski et al. (2020)	The results show notable differences in extraversion, agreeableness, and openness traits between LoL players of lower and higher ranks. Surprisingly, despite LoL being a team-oriented game, higher performance did not align with increased extraversion and agreeableness. In fact, players with lower ranks had notably higher scores in these traits than their higher-ranked counterparts
Piepiora and Piepiora (2021)	This study found that sports champions exhibit lower neuroticism and higher scores in the other Big Five personality dimensions compared to other athletes. Neuroticism was the key determinant for achievement levels. It is unclear whether these personality differences were shaped during athletes’ careers or existed from the beginning, suggesting that personality differences may be a consequence, rather than a cause, of athletes’ success
Zar et al. (2022)	The research highlighted a strong link between openness and athletes’ performance at the national level for both genders. In contrast, the relationships between neuroticism, extraversion, and conscientiousness with athletic performance varied widely across competitive tiers. Additionally, a significant connection was observed between agreeableness and performance at both provincial and national stages
Piepiora et al. (2021)	The passage highlights the importance of generating personality profiles of American football players in Poland and emphasizes the relevance of similar studies conducted in the United States. It also underlines the significance of defining personality in the recruitment of players to American football and suggests the need for further research on the relationship between personality and sports experience in all sports disciplines
Piepiora (2021a)	Significant differences were found in the personality traits of team sports players across different sports disciplines, except for openness to experience. This suggests that sports activity influences personality shaping, and that personality traits impact problem-solving in sports. The specificity of each sports discipline may impose slightly different psychological requirements on competitors. Other factors, such as previous experiences and social and cultural influences, should also be taken into account
Klatt et al. (2021)	Results indicated that compared to the norm, players demonstrated a higher level of neuroticism, but lower levels of extraversion, agreeableness, and conscientiousness. However, players exhibited traits of liveliness, tension, emotional stability, reasoning, and openness to change. Moreover, beach volleyball players showed well-established emotion regulation styles that allowed them to remain focused during matches
Fasold et al. (2019)	Performance level was notably related to the personality traits of conscientiousness and openness. However, no significant associations were found between performance and traits like extraversion, agreeableness, or emotional stability. Intriguingly, handball goalkeepers tended to be less receptive to new experiences than the general populace, yet displayed higher conscientiousness and neuroticism
Azita et al. (2019)	It was observed that neuroticism personality traits had a moderating effect on the impact of the psychological preparation program on the dimensions of sport performance, while other personality traits did not have a significant effect. These results highlight the importance of considering personality traits when designing and implementing psychological preparation programs for futsal players
Ruiz-Barquín and García-Naveira (2013)	Adult players showed higher emotional stability, openness to experience, and responsibility than juvenile players. Personality traits such as responsibility, openness to experience, and emotional stability were positively related to athletic performance. Regression models showed significant predictive capacity for neuroticism and openness to experience in the overall sample, and for neuroticism and responsibility in juvenile players and neuroticism and extraversion in players over 18 years old

In summary, this comprehensive review of 23 studies emphasizes the importance of accounting for personality traits in the recruitment and training of athletes, as these traits can influence problem-solving skills in sports and result in performance disparities.

## Quality assessment and analysis of publication bias

Table 3 displays an evaluation of the methodological integrity and potential bias present in the studies that were reviewed. By using the quality assessment of the 22 items in the STROBE statement, most of the analyses used technical terms in the title or abstract to describe the design of their studies (82.6%; *k* = 19). All studies presented the background of the survey, participant inclusion criteria, data sources, and evaluation methods for each study variable, and reported the results of the survey (100%, *k* = 23). Almost all surveys presented key elements of design (95.6%; *k* = 22) and outcomes that defined all predictors and potential factors (91.3%; *k* = 21) in the outcomes. Eighty-three percent (*k* = 19) of the studies listed clear goals and made prior assumptions. Eighty-seven percent (*k* = 20) of the studies referenced pre-listed objectives and summarized key results. 78.2% (*k* = 18) of the surveys described the setting of the study, location, and date, including recruitment, contact, follow-up, and time period for data collection, and gave information on participant characteristics (e.g., demographic, clinical, and sociological) and exposure and potential confounders. In addition, subgroup and interaction analyses and sensitivity analyses were reported in 73.9% (*k* = 17) of the findings. The results of 69.5% (*k* = 16) of the studies took objectives, limitations, diversity of analyses, results of similar studies, and other relevant evidence into account. 65.2% (*k* = 15) of the study data gave an unadjusted estimate, giving a confounder-adjusted estimate and its precision (e.g., 95% confidence interval). 60.8% (*k* = 14) of the studies described the statistical methods used, the number of individuals at each stage, and discussed the limitations of the study at the end of the results, taking into account potential sources of bias or imprecision. More than half of the studies explained how quantitative variables were treated in the analysis (56.5%; *k* = 13). Only 30% of the studies discussed the generalizability of the findings (*k* = 7). How the sample size was derived was explained in only 21.7% (*k* = 5) of the results. Only four studies (17.3%) described methods to address bias in the study and received funding.

**Table 3:** Quality assessment–individual evaluation of the studies examined.

References	STROBE Statement—Checklist of items that should be included in reports of cross-sectional studies																					
	1	2	3	4	5	6	7	8	9	10	11	12	13	14	15	16	17	18	19	20	21	22
Fabbricatore et al. (2021)	YES	YES	Partly	YES	Partly	YES	YES	YES	NO	NO	NO	Partly	YES	Partly	YES	YES	NO	YES	Partly	YES	Partly	YES
Ionel et al. (2022)	YES	YES	YES	YES	Partly	YES	YES	YES	Partly	NO	Partly	Partly	YES	YES	YES	YES	YES	YES	YES	Partly	Partly	YES
Siemon and Wessels (2022)	YES	YES	YES	YES	YES	YES	YES	YES	Partly	NO	YES	YES	YES	YES	YES	YES	YES	YES	YES	Partly	YES	NO
Piedmont et al. (1999)	YES	YES	YES	YES	YES	YES	YES	YES	Partly	NO	NO	NO	Partly	YES	YES	YES	YES	YES	YES	YES	Partly	NO
Lin et al. (2011)	Partly	YES	Partly	YES	Partly	YES	YES	YES	Partly	NO	NO	NO	NO	NO	YES	YES	YES	YES	Partly	YES	YES	NO
Liu (2011)	Partly	YES	YES	YES	YES	YES	YES	YES	YES	NO	YES	YES	YES	YES	YES	YES	YES	YES	YES	YES	YES	NO
Allen et al. (2011)	Partly	YES	YES	YES	YES	YES	YES	YES	YES	YES	YES	YES	YES	YES	YES	Partly	YES	YES	YES	YES	YES	NO
Zhu et al. (2013)	Partly	YES	Partly	YES	YES	YES	YES	YES	Partly	NO	Partly	NO	Partly	Partly	YES	YES	YES	NO	Partly	YES	YES	YES
Terracciano et al. (2013)	YES	YES	YES	YES	YES	YES	YES	YES	Partly	NO	YES	YES	Partly	YES	YES	YES	YES	YES	YES	YES	Partly	NO
Balyan et al. (2016)	YES	YES	YES	YES	YES	YES	YES	YES	Partly	NO	YES	Partly	NO	Partly	YES	YES	YES	YES	YES	YES	Partly	NO
Klein et al. (2017)	YES	YES	YES	YES	YES	YES	YES	YES	Partly	NO	YES	YES	YES	YES	YES	YES	YES	YES	NO	Partly	Partly	NO
Stine et al. (2019)	YES	YES	YES	YES	YES	YES	YES	YES	Partly	YES	YES	YES	Partly	YES	YES	YES	YES	YES	Partly	YES	Partly	YES
Yuan (2020)	YES	YES	YES	YES	YES	YES	YES	YES	Partly	NO	Partly	Partly	YES	YES	YES	YES	YES	YES	YES	YES	YES	NO
Kalinowski et al. (2020)	YES	YES	YES	YES	YES	YES	YES	YES	Partly	NO	YES	YES	YES	YES	YES	YES	YES	Partly	YES	Partly	Partly	NO
Matuszewski et al. (2020)	YES	YES	YES	YES	YES	YES	YES	YES	Partly	Partly	NO	NO	YES	YES	YES	YES	Partly	YES	YES	YES	Partly	NO
Piepiora and Piepiora (2021)	YES	YES	YES	YES	YES	YES	YES	YES	Partly	Partly	NO	YES	YES	YES	YES	NO	Partly	Partly	YES	Partly	Partly	NO
Zar et al. (2022)	YES	YES	YES	YES	YES	YES	YES	YES	Partly	NO	YES	YES	NO	Partly	YES	NO	YES	YES	NO	Partly	NO	NO
Piepiora et al. (2021)	YES	YES	YES	YES	YES	YES	YES	YES	YES	YES	YES	YES	YES	YES	YES	Partly	Partly	YES	Partly	Partly	Partly	NO
Piepiora (2021a)	YES	YES	YES	YES	YES	YES	YES	YES	Partly	NO	YES	YES	Partly	YES	YES	NO	Partly	YES	Partly	YES	Partly	NO
Klatt et al. (2021)	YES	YES	YES	YES	YES	YES	YES	YES	YES	YES	YES	YES	Partly	YES	YES	NO	YES	YES	YES	YES	Partly	NO
Fasold et al. (2019)	YES	YES	NO	YES	Partly	YES	Partly	YES	NO	NO	Partly	YES	YES	YES	YES	YES	Partly	YES	YES	YES	Partly	NO
Azita et al. (2019)	YES	YES	YES	YES	Partly	YES	Partly	YES	NO	NO	Partly	Partly	YES	YES	YES	Partly	YES	YES	Partly	YES	Partly	NO
Ruiz-Barquín and García-Naveira (2013)	YES	YES	YES	Partly	YES	YES	YES	YES	Partly	YES	YES	YES	YES	YES	YES	Partly	YES	YES	YES	YES	YES	NO

1 = Indicate the study’s design with a commonly used term in the title or the abstract; 2 = Explain the scientific background and rationale for the investigation being reported; 3 = State specific objectives, including any prespecified hypotheses; 4 = Present key elements of study design early in the paper; 5 = Describe the setting, locations, and relevant dates, including periods of recruitment, exposure, follow-up, and data collection; 6 = Give the eligibility criteria, and the sources and methods of selection of participants; 7 = Clearly define all outcomes, exposures, predictors, potential confounders, and effect modifiers. Give diagnostic criteria, if applicable; 8* = For each variable of interest, give sources of data and details of methods of assessment (measurement). Describe comparability of assessment methods if there is more than one group; 9 = Describe any efforts to address potential sources of bias; 10 = Explain how the study size was arrived at; 11 = Explain how quantitative variables were handled in the analyses. If applicable, describe which groupings were chosen and why; 12 = Describe all statistical methods, including those used to control for confounding.; 13* = Report numbers of individuals at each stage of study—e.g., numbers potentially eligible, examined for eligibility, confirmed eligible, included in the study, completing follow-up, and analyzed; 14* = Give characteristics of study participants (e.g., demographic, clinical, social) and information on exposures and potential confounders; 15* = Report numbers of outcome events or summary measures; 16 = Give unadjusted estimates and, if applicable, confounder-adjusted estimates and their precision (e.g., 95% confidence interval). Make clear which confounders were adjusted for and why they were included; 17 = Report other analyses done—e.g., analyses of subgroups and interactions, and sensitivity analyses; 18 = Summarize key results with reference to study objectives; 19 = Discuss limitations of the study, taking into account sources of potential bias or imprecision. Discuss both direction and magnitude of any potential bias; 20 = Give a cautious overall interpretation of results considering objectives, limitations, multiplicity of analyses, results from similar studies, and other relevant evidence; 21 = Discuss the generalizability (external validity) of the study results; 22 = Give the source of funding and the role of the funders for the present study and, if applicable, for the original study on which the present article is based. Yes = Available; Partly = Partly available; No = Not available.

## Discussion

This retrospective analysis provides the most comprehensive statistical review to date of the relationship between personality and athletic performance and confirms that the Big Five personalities are associated with athletic performance. It is clear that the Big Five personality model can provide a practical level of statistical prediction for socially important sports performance standards. Athlete personality traits can be used to predict athletic performance and provide direction for recruiting athletes and preparing for competition. Such as openness, agreeableness, and conscientiousness, which have been found to predict performance in specific sports (e.g., rock climbing, rowing), neuroticism appears to negatively affect sports performance, and lower levels of neuroticism are associated with higher performance (e.g., soccer, boxing, and martial arts). It is important to note that the personality characteristics of athletes in different sports may vary, highlighting the importance of considering the specific requirements of each sport (e.g., team sports, extreme sports). These findings may vary by sport, level of competition, and other factors such as cultural and social influences. Therefore, future researchers and scholars need further research to better understand the complex relationship between the five-factor model of personality and sports performance in different situations.

Individuals characterized by openness tend to appreciate diversity, pursue novel experiences, and exhibit curiosity and insight regarding their surroundings. These traits align with the characteristics required for rock climbing, which encompass both sport climbers and boulderers. A positive correlation has been observed between openness scores and climbing performance (Ionel et al., 2022). Risk-taking is frequently deemed a crucial subcomponent of openness, resulting in participants in high-risk sports demonstrating significantly elevated levels of extraversion and experiential openness (Tok, 2011). A robust predictive index exists between performance in Nanquan and openness to experience (Yuan, 2020). Similarly, openness is a vital attribute in American football. Consequently, systematic personality testing among athletes is advocated during the selection process for top-tier American football competitions (Piepiora et al., 2021).

A strong correlation exists between conscientiousness and team events. In assessments of football and basketball performance, conscientiousness demonstrates a positive association with sports achievements (Kalinowski et al., 2020; Siemon and Wessels, 2022). In team sports, individual performance substantially influences competition outcomes, indicating that traits such as self-control, diligence, responsibility, and reliability contribute to enhanced performance. In contrast, specific individual exercises, including swimming and aerobic activity, exhibit no relationship with conscientiousness, aligning with earlier research. The incremental validity of researchers’ incapacity to predict endurance athletes’ performance via conscientiousness and motivation has been substantiated (Perry et al., 2017). Notwithstanding that descriptors like “diligence,” “dependability,” and “persistence” encapsulate conscientious traits, and persistence appears essential for endurance sports, the present investigation reveals no association between these elements.

Extroversion, a personality dimension, is typically associated with individuals who exhibit talkativeness, confidence, and affability. In the realm of sports, extroverted athletes are capable of engaging in effective communication with teammates, coaches, and competitors, fostering a conducive atmosphere and enhancing performance. This advantage becomes particularly salient in team sports contexts. For instance, extroverted NBA players exhibit positive correlations with various performance metrics. Basketball athletes displaying pronounced extroversion are more capable of tolerating pain due to the inherent nature of team sports (Siemon and Wessels, 2022). These individuals relish camaraderie, support, and competition while striving toward shared objectives. This notion is substantiated by the mediating effect of extraversion on performance effectiveness in relation to the task-centered approach to stress among football players (Kalinowski et al., 2020). Interestingly, a survey of individual event champions also revealed markedly high extraversion traits (Piepiora, 2021b). This observation aligns with the proclivity of active individuals to exhibit traits such as energetic demeanors, fast-paced lifestyles, confidence, and positive emotions, all of which fall within the domain of extraversion. Moreover, extroversion has been linked to increased aerobic capacity (Terracciano et al., 2013).

Agreeableness, which focuses on qualities like trust, altruism, modesty, compassion, cooperation, and honesty, is distinct from extraversion. Athletes in team sports often score higher in agreeableness compared to those in individual sports. Given its socially-oriented nature, this correlation seems logical (Nia and Besharat, 2010). But studies in beach volleyball, basketball, and American football have not confirmed this result (Klatt et al., 2021; Siemon and Wessels, 2022), in contrast to individual sports such as canoeing. There is a significant relationship between agreeableness and sport performance in rowing, with elite athletes with high agreeableness having better performance (Liu, 2011; Stine et al., 2019).

Among the “Big Five” personality dimensions, neuroticism stands out as the sole attribute with an inherently negative implication. This trait is intrinsically linked to the autonomic nervous system’s excitation levels, with neurotic individuals possessing highly unstable systems characterized by rapid agitation onset and a prolonged return to baseline (Eysenck, 1967). A plethora of research findings demonstrate that athletes exhibiting lower neuroticism levels exhibit superior sports performance, with team sports champions displaying reduced neuroticism relative to their counterparts (Piepiora, 2021a). Additionally, elite athletes exhibit lower neuroticism levels compared to non-elite athletes (Vealey, 1992), and those participating in exercise display lower neuroticism scores than non-exercise participants (McKelvie et al., 2003). Concurrently, certain studies reveal that female athletes generally possess higher neuroticism levels compared to their male counterparts (Allen et al., 2011; Lin et al., 2011), though this observation is contextually specific to Nanquan (Yuan, 2020).

The relationship between personality and performance varies across athlete levels. Studies focusing on high-level athletes, such as those aiming for the NBA, elite canoeists, or national team members, frequently highlight traits like conscientiousness, neuroticism, and agreeableness as predictive of performance outcomes (Liu, 2011; Siemon and Wessels, 2022). Notably, low neuroticism and high conscientiousness often correlate with better performance in these elite settings. This is consistent with the results of a study conducted by Steca et al. (2018), who found that more successful athletes displayed higher levels of agreeableness, conscientiousness, and emotional stability compared to those who were less successful. In non-elite athletes, the Five Factor Model of personality shows a complex relationship with sports performance. Unlike elite counterparts with distinct profiles, these athletes exhibit a wide personality range, where traits like agreeableness can predict climbing prowess (Ionel et al., 2022), while neuroticism might influence outcomes in computer-based soccer (Balyan et al., 2016) and martial arts (Yuan, 2020). It’s imperative, as underscored by García Naveira (2010), Garcia-Naveira and Ruiz-Barquín (2020), and Piedmont et al. (1999), to make a clear distinction between studies. Some research focuses on disparities grounded in the athletes’ competitive tiers, while others delve into the intricate relationships between personality traits and performance metrics.

Other studies have shown a correlation between personality traits and energy expenditure in elderly individuals, and found a significant correlation between neuroticism, extroversion, openness, and conscientiousness with energy expenditure during peak walking speed (Terracciano et al., 2013). But this is contrary to the latest study, Personality traits did not moderate intervention effects on physical functioning (Kekäläinen et al., 2023). It’s evident that the influence of personality on performance can be multifaceted and may vary based on the sport, the competitive tier, and individual factors. When considering differences among sports, athletes in high-risk sports may be characterized by their thrill-seeking nature and openness to experiences. Meanwhile, those in team sports, like soccer, often display more extroversion but may be less emotionally stable compared to individual sport athletes. Furthermore, team sport participants tend to balance personal and group needs, whereas those in individual sports lean more toward individualism (García Naveira, 2010). In essence, the intricate interplay between personality and performance underscores the importance of considering both the individual’s psychological makeup and the unique demands of their chosen sport when evaluating and predicting athletic success.

## Study strengths and limitations

By compiling and analyzing an extensive array of pertinent literature, this review offers a thorough and insightful examination of the associations between the five personality traits and athletic performance. The investigation encompasses not only the overarching connections between these traits and performance but also delves into the impacts of various sport types, athletic populations, and athletes’ ages on these relationships. The findings hold substantial relevance for sports psychologists, coaches, athletes, and sports administrators, furnishing valuable guidance for selection processes, training, and psychological interventions.

However, the comprehensive nature of the research domains addressed and the diverse characteristics of sports and populations could introduce heterogeneity into the study, potentially affecting the results’ robustness and precision. Additionally, distinct cultural backgrounds may exert differential influences on personality traits and athletic performance, thereby constraining the applicability of the findings across diverse cultural contexts. In summary, while this review sheds light on the interplay between the five personality traits and sports performance, further meticulous and exhaustive research is necessary to surmount existing limitations and furnish more reliable evidence for practitioners.

## Recommendations for future research

We suggest the following directions for future research: Utilize longitudinal study designs to establish causal relationships between the Big Five personality traits and athletic performance more effectively. Perform cross-cultural investigations to assess how the connections between personality traits and sports performance might differ in various cultural settings, thereby increasing the applicability of research findings. Examine the relationships between personality traits and athletic performance in specific sports and populations, such as team sports, individual sports, aerobic sports, and among professional, amateur, and youth athletes, offering tailored guidance for practitioners. Evaluate the impact of targeted psychological interventions focusing on personality traits in enhancing sports performance, assisting sports psychologists and coaches in designing effective approaches. Apply advanced statistical methods like structural equation modeling and multilevel analysis to deepen our understanding of the relationships between personality traits and sports performance while accounting for potential interactions and moderating variables. Investigate the interplay between personality traits and other psychological aspects, including motivation, self-efficacy, and emotion regulation, to gain a more comprehensive perspective on the psychological mechanisms driving sports performance. By addressing these suggestions, future research can contribute to a richer understanding of the links between the Big Five personality traits and athletic performance, ultimately providing valuable support for practitioners in the field.

## Conclusion

This systematic analysis reveals that the relationship between personality and sports performance is influenced by various factors, including the type of sport, the athletic population, and the athletes’ age. The analysis indicates that, aside from neuroticism, openness, conscientiousness, extraversion, and agreeableness display positive correlations with sports performance. Conscientiousness and extraversion emerge as predominant personality traits in team sports, contributing to team cohesion and, ultimately, improved outcomes. The influence of openness and agreeableness varies across different sports, with no definitive consensus on the specific sports they benefit. Overall, the findings offer promising insights into the potential of personality as a predictor of athletic performance. As a result, stakeholders such as applied sports psychologists, coaching staff, athletes, and sports administrators must adequately comprehend the role of personality and its pertinence to success in major competitions. Practitioners should advocate for the integration of personality assessment and development programs within the training process. To achieve this, a more profound understanding and utilization of personality concepts in physical education and sports are required, along with the development of effective, user-friendly personality tools and well-founded, easily implemented training programs.

## Author contributions

YS: Conceptualization, Data curation, Formal analysis, Methodology, Visualization, Writing – original draft, Writing – review & editing. SW: Conceptualization, Data curation, Visualization, Writing – original draft, Writing – review & editing. XL: Conceptualization, Funding acquisition, Visualization, Writing – original draft, Writing – review & editing. YK: Conceptualization, Data curation, Formal analysis, Supervision, Writing – original draft, Writing – review & editing. GK: Conceptualization, Data curation, Formal analysis, Supervision, Writing – original draft, Writing – review & editing.
